# Co-Creating a Digital Resource to Support Smartwatch Use in COPD Self-Management: An Inclusive and Pragmatic Participatory Approach

**DOI:** 10.3390/healthcare14010037

**Published:** 2025-12-23

**Authors:** Laura J. Wilde, Louise Sewell, Nikki Holliday

**Affiliations:** 1 Centre for Healthcare and Communities, Coventry University, Coventry CV1 5RW, UK; nikki.holliday@coventry.ac.uk; 2 School of Life Course & Population Sciences, Faculty of Life Sciences & Medicine, King’s College London, London SE1 1UL, UK; 3 School of Health and Care, Coventry University, Coventry CV1 5RW, UK; louise.sewell@coventry.ac.uk

**Keywords:** smartwatches, co-creation, digital health, respiratory disease, self-management

## Abstract

**Highlights:**

**What are the main findings?**

- A co-created digital resource (website and video) was developed with people with COPD, carers, and healthcare practitioners to support smartwatch use for COPD self-management.
- Participants identified key needs, including understanding smartwatch features, interpreting health data, and accessing relatable, practical guidance.

**What are the implications of the main findings?**

- The resource offers tailored support for both patients and practitioners, with potential to enhance everyday use of wearable technologies in COPD care.
- The innovative, rigorous, and inclusive co-creation process ensured the resource was relevant, acceptable, and grounded in lived experience.

**Abstract:**

Wearable technologies, such as smartwatches, are increasingly used by people with Chronic Obstructive Pulmonary Disease (COPD) for health monitoring and self-management. However, there is limited evidence-informed guidance available to help patients and healthcare practitioners use these tools effectively in everyday life. **Objectives:** This study aimed to co-create a digital resource for people with COPD and healthcare practitioners to support the use of smartwatches for self-management. **Methods:** A participatory co-creation methodology was used, based on the Three Co’s Framework (co-define, co-design, co-refine). Participants included people with COPD, carers, family, or friends of people with COPD; healthcare practitioners; and researchers who attended workshops and individual think-aloud interviews to develop a website and video resource. The resource was refined based on real-time feedback. Data were analysed using rapid qualitative analysis. **Results:** Twenty-one participants engaged and identified key informational needs, including understanding smartwatch features, interpreting health data, and setting personalised goals. The co-created website and video resource were positively received. Participants valued the inclusion of real-life experiences and practical guidance tailored to both patients and healthcare practitioners. **Conclusions:** This study presents the first co-created resource for COPD and healthcare practitioners on using smartwatches. The co-creation process was successfully delivered online and face-to-face, demonstrating a robust, inclusive approach to managing multiple stakeholders. The resource offers practical value for patients and practitioners and contributes to the growing field of remote interventions for chronic respiratory conditions. Future research is needed to evaluate its effectiveness.

## 1. Introduction

Chronic Obstructive Pulmonary Disease (COPD) is a progressive respiratory condition affecting over 300 million people globally and is the fourth leading cause of death worldwide [1]. In the UK, COPD contributes to over one million hospital bed days annually, placing a significant burden on individuals, families, and healthcare systems [2]. Effective self-management is essential to improve quality of life and reduce exacerbations and hospital admissions.

As healthcare systems increasingly adopt remote and digital approaches to chronic disease management, wearable technologies such as smartwatches offer new opportunities to support self-monitoring, symptom awareness, and personalised care outside of traditional clinical settings [3]. Smartwatch devices can track physical activity, heart rate, oxygen saturation, and sleep patterns, offering real-time feedback and promoting patient engagement [4]. In this study, we use the term smartwatches to refer to commercially available wrist-worn devices that combine activity tracking with health monitoring features. These are a subset of wearable technologies, which also include fitness bands and medical-grade monitors. Remote patient monitoring refers to the broader use of digital tools to collect and transmit health data from patients to healthcare providers [5].

Evidence suggests that digital health interventions using wearable devices can improve physical activity levels and health-related quality of life in people with COPD [6,7]. Activity monitors may also support goal setting, self-monitoring and understanding symptom patterns [8,9,10,11,12]. However, many studies have focused on short-term use within research settings, with limited evidence of sustained impact in routine care [11]. This gap between efficacy (benefits observed in controlled trials) and effectiveness (real-world impact) is often attributed to implementation challenges, including a lack of tailored guidance and limited integration into clinical practice [13].

Recent reviews have also highlighted the broader role of everyday and assistive technologies in supporting daily life activities for people with COPD, including social participation and independence [14]. Remote patient monitoring, including the use of smartwatches and activity monitors, is increasingly used in home settings and offers potential for early detection of exacerbations and improved chronic disease management [5,15]. People with COPD have expressed interest in using smartwatches and apps to monitor symptoms and vital signs but report uncertainty about how to interpret health data [15,16,17]. Studies suggest that these tools can empower patients to take a more active role in their care, prompting early intervention and enhancing engagement in daily activities [18,19]. Healthcare practitioners similarly recognise the potential of wearable technologies but often lack the training or institutional support to use them confidently in practice [20].

While smartwatches have shown promise across various long-term conditions, including cardiovascular and mental health disorders, challenges remain in implementing these devices in healthcare settings, particularly for pulmonary populations [13,21]. As health-monitoring features become standard in consumer-grade smartwatches, there is growing recognition of their potential to support COPD self-management in line with national strategies [7,19]. Despite growing interest in wearable technologies, there remains a lack of accessible, evidence-informed resources to guide their use in COPD self-management.

This study aimed to co-create a digital resource with stakeholders (e.g., people with COPD, friends, family, carers and healthcare practitioners) to support the use of smartwatches for self-management for people with COPD and healthcare practitioners. The research question was what digital resource do stakeholders (people with COPD, carers, and healthcare practitioners) want to co-create to support the use of smartwatches for COPD self-management in everyday life, and what evidence-informed content should be included to ensure its relevance, usability, and trustworthiness?

## 2. Materials and Methods

### 2.1. Study Design and Theoretical Framework

This study employed a participatory qualitative design using a co-creation methodology structured around the Three Co’s Framework, Co-define, Co-design and Co-refine [22], a recognised approach for participatory intervention development applied in chronic health populations [23]. The study was also guided by the participatory principles of inclusivity, power-sharing, and responsiveness [24]. These principles informed our recruitment strategy, workshop facilitation, and ethical considerations, ensuring that the process was not only methodologically sound but also equitable and responsive to participant needs.

A critical realist ontology and pragmatist epistemological approach underpinned the research, acknowledging the value of multiple stakeholder perspectives in developing interventions suited to real-world contexts.

### 2.2. Recruitment and Participants

Participants were recruited through social media advertisements (e.g., Twitter/X and relevant COPD groups with admin approvals), local relevant health charities (e.g., Asthma and Lung UK and Breathe Easy groups), and contacts or networks of the researchers. Eligibility criteria included being aged 18 or older, residing in the UK and having a personal or professional interest in COPD (Table 1).

Recruitment materials included a JISC Online Survey link and QR code with participant information about the research study and a consent form. Initial screening questions were included in the survey to ensure participants were eligible to participate.

We aimed to recruit 6–8 participants per workshop, and participants could take part in as many or as few workshops as they would like. Participants were offered £25 shopping vouchers as a thank you for their time.

### 2.3. Co-Creation and Data Collection Phases

**Figure 1:**
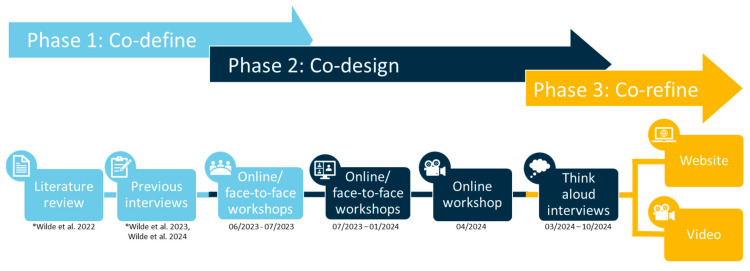
Phases of co-creation [11,15,20].

Data were collected across three phases, co-define, co-design, and co-refine, with a series of workshops (online and face-to-face) and one-to-one think-aloud interviews. Activities were designed to explore needs, develop content, and iteratively refine a digital resource to support smartwatch use in COPD self-management. Conversations during workshops and interviews naturally moved to the following stages (e.g., from co-define to co-design), hence the process and timeline are illustrated in Figure 1 with overlapping phases.

Workshops were facilitated by LW and NH while one-to-one interviews were conducted by LW. Microsoft 365 Bookings (Microsoft Corporation, Redmond, WA, USA). was used for interview scheduling. All sessions were audio/video-recorded using Zoom (Zoom Communications, San Jose, CA, USA), Microsoft Teams (Microsoft Corporation, WA, USA), or a Dictaphone (Sony ICD-AX412F). Recordings were transcribed using closed captioning via the video conferencing software or in Microsoft Word using the transcribe feature. Photographs and screenshots of the workshops were taken.

#### 2.3.1. Phase 1: Co-Define

The co-define phase aimed to explore the needs, concerns, and preferences of people with COPD and healthcare practitioners regarding smartwatch use in everyday life. This phase informed decisions about the format and tone of the resource (e.g., leaflet vs. website).

- Workshop 1 (06/2023) was conducted online using Zoom Whiteboard, allowing participants to express ideas visually and verbally (see Supplementary Materials S1).
- Workshop 2 (07/2023) was held face-to-face at a local pulmonary rehabilitation programme using paper-based tools (see Supplementary Materials S2).

#### 2.3.2. Phase 2: Co-Design

The co-design phase focused on collaboratively developing and prioritising the content, structure, and delivery format of the resource.

- Workshop 3 (07/2023) was conducted online using Zoom Whiteboard to prioritise content and format (see Supplementary Materials S3).
- Workshop 4 (01/2024) was held face-to-face and focused on developing content specifically for healthcare practitioners. Participants reviewed a printed version of the draft website and provided feedback on what information would be most useful in clinical settings.
- Workshop 5 (04/2024) gathered feedback on a draft version of the video component.

#### 2.3.3. Phase 3: Co-Refine

The co-refine phase involved one-to-one think-aloud interviews [25] to test and refine the draft website content (See Supplementary Materials S4 for the interview schedule). Think-aloud interviews involved participants verbalising their thoughts while navigating the resource, enabling real-time feedback on usability and content. Participants were invited to review the resource in real time and suggest edits.

- Participants were given the option to either view a screen-shared version of the draft or edit a live Word document themselves. Three chose the screen-share option, which was used consistently thereafter. The researcher used tracked changes to make edits and document iterative updates ready for the next participant. A table of changes was maintained to ensure transparency.
- During interviews, participants navigated the website prototype, developed (by LW) using WordPress (Automattic Inc., San Francisco, CA, USA), while sharing their screen. This allowed the researcher to observe user journeys and gather feedback on layout, language, and usability.
- In some cases, the researcher made live edits in WordPress to demonstrate changes and gather immediate reactions.
- Participants were also offered the option to test the website independently and provide feedback via email.

### 2.4. Data Analysis

Rapid qualitative analysis was selected to enable real-time, iterative development of the resource within time constraints while maintaining systematic coding and team validation.

Rapid analysis was conducted using thematic grouping at each phase on areas for development informing the next workshop/interview. Analysis was conducted by LW in consultation with NH through regular meetings and iterative discussion to agree the final themes. This analysis was a flexible and systematic approach to facilitate quick analysis and collaboration with co-creators throughout the co-creation phases. The themes for each phase of development are presented in the results.

### 2.5. Reflexivity

The lead researcher (LW) is experienced in qualitative research on COPD and wearable technologies. Reflexive notes were maintained throughout the study, and regular team meetings supported critical reflection. Participant input was highly valued and shaped the resource development.

### 2.6. Ethical Approval and Considerations

Ethical approval was obtained from the Coventry University Ethics Committee (Reference Number: P149985, 24 March 2024). Verbal and written informed consent for taking part in the research study, the use of participants’ data, and recording the interview was obtained prior to the workshops and interview via JISC Online Surveys. Interview transcripts were anonymised using participant numbers. Participant demographics are reported in aggregate form to preserve anonymity.

## 3. Results

### 3.1. Participants

Thirty-four participants consented to take part in the research study. Of those, 21 took part in at least one workshop or interview (Table 2). Participants who did not take part were either unavailable for workshops or did not respond to invitations to participate in interviews. One participant was excluded due to not residing in the UK. Two participants withdrew from the study in the co-define phase; one participant with COPD consented to their data being retained, while the other requested full withdrawal, and their data were deleted in accordance with ethical protocols.

Workshops lasted approximately 1 h 30 min and think-aloud interviews lasted between 24 min and 1 h 48 min (*M* = 1 h 6 min).

Of the participants who engaged with the research study, 8 were ‘male’, 11 ‘female’ and one ‘transgender’ (see Table 3). Participants’ ages ranged from 24 to 69 years at the time of consent (*M* = 45 years). Four participants had COPD, three were a friend, family or carer of a person with COPD, 11 were working as a healthcare practitioner (i.e., physiotherapist, occupational therapist, clinical exercise physiologist, nurse practitioner, doctor), two were researchers, and one was a respiratory charity group organiser. Healthcare practitioners reported working in their profession for between 2 and 30 years (*M* = 14 years). Participants with COPD reported being diagnosed from 2005 to 2020. Participants with COPD reported MRC Dyspnoea Scale Grade 0 (*n* = 1), Grade 1 (*n* = 1), and Grade 3 (*n* = 2). Three participants with COPD reported they had engaged with pulmonary rehabilitation, and three used inhalers.

Fourteen participants, including three participants with COPD, reported that they were currently engaging with mobile phone apps or wearables to track their activity (e.g., Apple watch, Samsung Galaxy watch, Fitbit watch, and Garmin watch), and four participants were not. One participant with COPD who was not engaging with activity monitors at the start of the study reported that during the study they were gifted a smartwatch from their family.

### 3.2. Development Phases and Analysis

The co-define, co-design, and co-refine were not always discussed linearly, with some phases overlapping (as demonstrated in Figure 1). The findings are presented in line with the co-creation phases.

#### 3.2.1. Phase 1: Co-Define—Identifying Needs and Priorities

The co-define phase involved two workshops (online and face-to-face) focusing on defining the needs, concerns and expectations of people with COPD and healthcare practitioners around the use of smartwatches for self-management in everyday life. Four themes were developed from this phase.

Theme 1.1: Uncertainty and knowledge gaps

Participants expressed a lack of clarity about what smartwatches could do, how they worked, and whether they were suitable for people with COPD. This uncertainty was shared across stakeholder groups and reflected broader concerns about digital health literacy and the absence of accessible guidance.

We need to be informed on what these devices can actually do so that we can appropriately advise.

(P1, Workshop 1)

I think you need to do something that would be creating some education materials for healthcare professionals, so that they can become aware of what wearables can do and how they might help.

(P5, Workshop 1)

This lack of knowledge was not simply technical; it extended to understanding how smartwatch data could be interpreted in the context of COPD symptoms and whether such data could be trusted or used meaningfully in clinical conversations.

Theme 1.2: Emotional responses to health data

Participants described how real-time health metrics, particularly oxygen saturation and heart rate, could provoke anxiety, especially during exacerbations or periods of breathlessness. While some found the data reassuring, others felt overwhelmed or confused by readings that appeared abnormal but were not clinically concerning.

So, I suppose it’s giving them the knowledge isn’t it… Then you don’t want them to worry if [oxygen saturation] is just from your finger, some of them all look at their watch and they’ll panic like “Oh my God, it’s below 90. Ohh I’m not OK” and like instantly panic. And actually with their condition it’s not uncommon that it’s below.

(P14, Workshop 2)

This highlighted the need for contextualised information that could help users interpret data safely and avoid unnecessary worry. Participants suggested that the resource should include clear explanations of what different metrics mean and when to seek help.

Theme 1.3: Desire for peer support and relatability

Participants emphasised the importance of hearing from others with lived experience. Peer stories were seen as a way to normalise the use of technology, reduce isolation, and build confidence. This was particularly important for people with COPD who felt uncertain about whether smartwatches were ‘for them.’

When you’ve got people’s experiences, it makes you feel a little bit more comfortable that you’re not out there on your own.

(P9, Workshop 3)

Participants suggested that the resource should include real-life examples, quotes, and videos from people with COPD to make the information more relatable and trustworthy.

Theme 1.4: Practical barriers and the importance of conversation

Time constraints, lack of training, and limited access to devices were identified as barriers to effective use. Healthcare practitioners noted that supporting patients with technology often relied on their own initiative rather than formal guidance or institutional support.

It’s just a bit more time, isn’t it? Because you would spend more time with people going through it really…

(P15, Workshop 2)

I think conversation is the most important thing, isn’t it?

(P14, Workshop 2)

Participants highlighted the importance of dialogue between patients and practitioners, not only to explain how devices work, but to explore how they could be used meaningfully in everyday life. This theme informed the inclusion of conversation prompts and shared decision-making tools in the final resource.

*Reflections and development decisions*

The co-define phase revealed a clear need for a resource that was informative and emotionally supportive, grounded in real-life experiences. Participants’ emphasis on uncertainty, anxiety, and the value of peer stories directly shaped the tone and priorities of the resource. These insights led the research team to prioritise clarity, relatability, and reassurance in the next phase. The decision to include real-life quotes and examples throughout the resource was a direct response to this early feedback. Additionally, the recognition of practical barriers prompted the team to explore formats that could be accessed easily and shared in clinical settings.

#### 3.2.2. Phase 2: Co-Design—Shaping the Format and Content

The co-design phase involved two workshops (one online and one face-to-face) and focused on collaboratively shaping the format, tone, and structure of the digital resource. Participants prioritised content, suggested delivery formats, and reflected on how the resource could meet the needs of both people with COPD and healthcare practitioners. Four themes were developed.

Theme 2.1: Format preferences and accessibility

Participants discussed various delivery formats, including printed leaflets, smartphone apps, and websites. While some initially favoured leaflets for their familiarity, most participants ultimately preferred a website due to its accessibility, flexibility, and potential for regular updates. Leaflets were seen as static and less engaging, particularly for conveying interactive or personalised content.

I think the leaflet is probably … the last thing you need.

(P14, Workshop 2)

I feel like this type of information could still be on the Asthma and Lung UK website … then it’s in that category of general information for everyone.

(P3, Workshop 3)

The preference for a website reflected a desire for a centralised, trusted source of information that could be accessed by both patients and practitioners. Participants also noted that a website could be more easily shared in clinical settings and linked to existing resources.

Theme 2.2: Visual and audio learning preferences

Participants expressed a strong preference for visual and auditory formats, particularly video. Videos were seen as ‘better than just text’ and more engaging, easier to understand, and better suited to demonstrating practical tasks such as setting up a smartwatch or interpreting health data.

Video’s the way forward with this…

(P4, Workshop 3)

This feedback informed the development of a short video to accompany the website. Participants suggested that videos could be hosted on YouTube and/or embedded within the website to maximise reach and accessibility.

Theme 2.3: Balancing simplicity and functionality

While most participants supported the idea of a website, some expressed interest in a smartphone app that could be downloaded and used offline. This highlighted a tension between simplicity and functionality; participants wanted the resource to be easy to use, but also capable of supporting personalised and on-the-go access.

I would prefer an app … it’s more accessible if it’s on my phone… If I needed to know how to use something to do with my wearable and I’m out, then it’s much more accessible.

(P5, Workshop 3)

This feedback was considered during development, but the decision to prioritise a website was based on feasibility, cost, and the ability to update content regularly. The website was designed to be mobile-friendly and accessible across devices.

Theme 2.4: Prioritising Patient Needs First

There was consensus that the resource should be developed primarily for people with COPD, with a separate section for healthcare practitioners added later. Participants felt that patients needed clear, relatable information first, and that practitioner content should support, rather than dominate, the resource.

Start with the patient side … then build the practitioner bit around it.

(P14, Workshop 3)

*Reflections and development decisions*

The co-design phase was built directly on the priorities identified in co-define. Participants’ preference for a website over a leaflet or app reflected a desire for accessibility and trustworthiness. Their emphasis on visual learning led to the development of a video component, and their feedback on sequencing ensured that the resource remained patient-centred. The research team reflected on the tension between simplicity and functionality and made pragmatic decisions to prioritise a mobile-friendly website that could be updated regularly. These decisions were shaped not only by participant input but also by feasibility and sustainability considerations.

#### 3.2.3. Phase 3: Co-Refine—Iterative Testing and Feedback

Participants provided detailed feedback through one-to-one think-aloud interviews and email feedback on the resource, leading to refinements in layout, language, and content; four themes were developed. Most suggestions from think-aloud interviews were incorporated with minimal contradictions.

Theme 3.1: Formatting and design

Suggestions for changes to the design and formatting included adjusting image alignment, changing the page layout, making important text and phrases bold, and including more diverse imagery.

Maybe even having bold on the specific sentence within the paragraph that is the actual point.

(P3)

I wonder if we could change the photo here for an inhaler…

(P33)

Theme 3.2: Navigation and user journey

Most found the site easy to use and navigate during the user testing. The researcher asked participants to use the website as they usually would; however, most navigated through it systematically as they did not want to miss anything.

That’s [navigation] really easy. Even just like the drop-down boxes, they come down so easy and so quick … Everything so well laid out and so easy.

(P18)

Nevertheless, participants also suggested adding or taking away links to support the navigation between the ‘for patients’ pages and the ‘for healthcare practitioners’ pages.

Yeah, but then I suppose at the same time, you could also link them that to that, that page, the patient page.

(P14)

Theme 3.3: Missing content and suggestions

Participants requested more information on interpreting health metrics, setting step goals, and managing sedentary behaviour. For example, to include ‘normal’ or ‘safe’ oxygen saturation levels and heart rate levels.

I think it’s worth adding as well their heart rate … Well, it’s just heart rate monitoring.

(P2)

Participants also suggested including information about physical activity step goals, energy levels and pacing.

Yeah, yeah. Knowledge that you may plateau at the same level so, you know, sometimes maintaining is a win. Yeah, that’s probably what I would put there …

(P20)

It’s useful to put it in there. Maybe if you can’t handle your step goal in one go to do two sessions, one in the morning and one in the evening, or break it down into bite sized chunks.

(P4)

Theme 3.4: Rewording and refining language

The language and wording of the information were particularly important to participants. Participants preferred direct, supportive language and suggested rewording technical terms.

How? What can smartwatches do to help with COPD management? I wonder whether about using the word ‘you’ in there. Actually, ‘how’ can smartwatches help ‘you’ manage your COPD, so it becomes a bit more direct?

(P20)

OK, I wouldn’t call it ‘breathing exercises’. I will tell you why. But but that’s me that I’m very particular about this because we are not exercising anything. If they are like breathing techniques, I will call it, and perhaps not all are effective, not all are actually good, but it’s like you could mention one technique which got some evidence is called pursed breathing.

(P33)

As changes were made, feedback from participants was consistently positive, as reflected in participant quotes describing the resource as ‘easy to navigate’ and ‘useful’.

No, it’s it’s good. It’s good, I think it’s. I think it’s a good start. You know, it’s to start the conversation as well between the healthcare professionals and patients. And you know and give the information because it’s like it may be easier for healthcare professionals to to, you know, to give to have a place to go to, to start these conversations.

(P33)

*Reflections and development decisions*

The co-refine phase demonstrated the value of iterative feedback and real-time collaboration. Participants appreciated being able to see their suggestions actioned during interviews, which fostered a sense of ownership and trust. The refinements made during this phase addressed core concerns raised in earlier phases, such as emotional safety, accessibility, and relevance. The resource evolved from a draft document into a fully functioning website shaped by lived experience, professional insight, and collaborative design.

Dissemination of the resource was guided by participant suggestions, including the use of webinars and YouTube videos. Participants’ input shaped the format and priorities of the website’s online launch event held in November 2024 [26].

## 4. Discussion

This study presents a novel, co-created digital resource, comprising a website [27] and video to support remote self-management among people with COPD and to guide healthcare practitioners in using smartwatches as part of COPD care. Developed through a rigorous participatory process, involving patients, carers, and healthcare practitioners, the resource addresses a critical gap in accessible, evidence-informed guidance on wearable technology use in chronic respiratory disease. The resource functions as a remote support tool that can be used independently by patients or integrated into consultations, pulmonary rehabilitation, or telehealth services which may support self-management interventions.

Our findings align with previous research highlighting the potential of wearable technologies to support self-management in COPD [7,16]. Similarly to Lundell et al. [28] and Kjellsdotter et al. [29], this study demonstrates the value of co-creation in developing digital health interventions that are acceptable and relevant to end users. However, our study extends this work by explicitly addressing the needs of both patients and healthcare professionals, and by focusing on the integration of wearable technology into everyday life.

### 4.1. Strengths and Limitations

A key strength of this study is its rigorous co-creation process and methodological approach, guided by the Three Co’s Framework [22] and the principles articulated by Leask et al. [24]. The co-creation process successfully incorporated perspectives of multiple stakeholders, including people with COPD and healthcare practitioners, through a combination of online and in-person workshops, complemented by one-to-one think-aloud interviews. The robust, inclusive and iterative design ensured a bottom-up approach, responsiveness to participant feedback, and produced a resource grounded in lived experience and clinical insight.

However, there are limitations to consider. While the resource was co-created with input from COPD patients and healthcare practitioners, the sample may not fully represent the broader population affected by COPD, especially given ongoing challenges related to digital literacy and device accessibility [16]. Recruitment via social media may have introduced bias toward digitally literate individuals and COPD diagnosis was self-reported and not clinically verified, which may limit clinical validity. However, the resource was intended for those interested in or already using technology in everyday life, rather than advocating device purchase. Also, despite extensive recruitment efforts, only four participants with COPD took part; however, they were highly engaged across multiple workshops and interviews, contributing substantively to the development and refinement of the resource. Participants with COPD had a strong and clear voice throughout the study. Their input was prioritised and instrumental in shaping the patient-facing content and ensuring its relevance and accessibility. Healthcare professionals’ views were positioned as supportive rather than dominant.

Separate workshops were conducted for HCPs and people with COPD due to scheduling and relevance of content. While this limited cross-group dialogue, it allowed participants to focus on their respective needs. Mixed-group sessions, such as video development, were successful and demonstrated collaboration and alignment across stakeholder perspectives. The inclusion of a variety of healthcare professions was essential to ensure the resource was scientifically accurate, ethically sound, and safe for implementation. Their input helped mitigate potential risks and ensure the resource could be confidently used.

### 4.2. Implications for Future Research and Clinical Practice

This resource offers practical value for people with COPD and healthcare professionals supporting people with COPD. With increasing smartwatch adoption, this resource provides timely support to help people with COPD use this technology to maximise self-management. The website can be used in consultations to introduce smartwatch use, explain key features, and guide patients in interpreting health data. The inclusion of real-life examples and conversation prompts may facilitate shared decision-making and enhance engagement. However, potential risks identified during co-creation include cognitive burden when interpreting health metrics, which may require additional support or simplified guidance tailored to specific devices to avoid confusion or anxiety. Further research is needed to assess its effectiveness in improving self-management outcomes. 

This research to co-create a resource for people with COPD and healthcare practitioners opens possibilities for further study on how digital health interventions can better serve populations with COPD, particularly those with limited access to pulmonary rehabilitation [4]. Future research could evaluate the effectiveness of such resources and assess how digital self-management support impacts health outcomes over time [7]. There is also potential to explore additional features on the website, such as interactive modules or integration with telehealth services, which could expand the scope of self-management for COPD patients [16].

While this co-created digital resource represents advancements in supporting COPD self-management through smartwatch technology, it is important to consider the rapidly evolving landscape of digital health. As wearable technology continues to develop, the integration of sophisticated sensors, improved algorithms, and artificial intelligence holds promise for revolutionising COPD care [5]. However, further research is needed to establish clinical efficacy and guide widespread adoption.

In line with the World Health Organization’s global strategy to expand and strengthen digital health implementation and self-management resources, this website provides much-needed support [30]. Despite ongoing barriers to digital health access, including limited device ownership and digital literacy among older adults, this study marks a step forward in supporting COPD self-management [4]. The resource offers a valuable foundation for guiding patients and healthcare practitioners in understanding and leveraging smartwatch technology, moving closer to a more inclusive and effective model of digital healthcare for COPD [7]. By providing easily accessible information on using smartwatches for health monitoring, the website serves as an inclusive platform to empower both patients with COPD and healthcare practitioners, bridging the gap between technology and practical application in COPD management.

## 5. Conclusions

This study presents the co-creation of a digital resource to support smartwatch use for people with COPD and guide healthcare practitioners in the use of smartwatches for COPD care. The resource addresses a gap in accessible, evidence-informed guidance and offers tailored content for both patients and practitioners. The study demonstrates how co-creation can be used to develop relevant, usable, and acceptable digital health tools. The findings highlight the importance of integrating lived experience and clinical insight into the design of self-management resources. The resource has the potential to support shared decision-making, improve confidence in using wearable technologies, and facilitate more personalised care. Future research should evaluate its impact on self-management behaviours, clinical outcomes, and practitioner engagement, and explore opportunities for broader implementation and adaptation across other long-term conditions.

## Figures and Tables

**Table 1:** Participant inclusion and exclusion criteria.

Inclusion Criteria	Exclusion Criteria
Aged 18 years or older UK-based resident Self-reported diagnosis of COPD (health records were not checked or the diagnosis verified), or friend/family/carer of someone with COPD, or healthcare professional involved in the care of people with COPD Able to give informed consent Able to communicate in English, to participate in the research activities Stable internet connection and an internet-enabled device (if accessing online workshops)	Severe, active mental health problems or diagnosis of dementia or other neurodegenerative disorder that may undermine person’s capacity to provide informed consent.

**Table 2:** Workshop and interview attendances.

Participant Number *, Role (*n* = 21)	Co-Define		Co-Design			Co-Refine		
	Workshop 1 (06/2023)	Workshop 2 (07/2023)	Workshop 3 (07/2023)	1-1 Interviews	Workshop 4 (01/2024)	Workshop 5 (04/2024)	1-1 Interviews	Feedback via Email
1, HCP	X							
2, HCP				09/2023				
3, Friend	X		X	09/2023			08/2024	X
4, COPD	X		X	10/2023		X	03/2024	
5, COPD	X		X					
9, COPD			X	11/2023		X	10/2024	
12, HCP		X			X	X		
14, HCP		X			X	X		
15, HCP		X			X			
16, HCP		X			X	X		
18, HCP							09/2024	
21, COPD				12/2023		X		
22, HCP							09/2024	
24, HCP						X	05/2024	
26, Charity							04/2024	
27, Friend							05/2024	
28, HCP							08/2024	X
32, Friend								X
33, HCP							10/2024	
34, Researcher								X
35, Researcher								X

* participant numbers of those who engaged with a workshop or interview. HCP: healthcare practitioners, COPD: Person with chronic obstructive pulmonary disease.

**Table 3:** Participant demographic details.

Demographic	Age in years, Mean (Range)	Gender
Friend, family or carer (*n* = 3)	33.7 (31–38)	1 female, 2 male
People with COPD (*n* = 4)	60.5 (38–69)	1 female, 2 male, 1 transgender
Healthcare practitioners (*n* = 11)	41.9 (24–63)	8 female, 3 male
Researchers (*n* = 2)	38.5 (27–50)	2 female
Charity representative (*n* = 1)	67	1 male

## Data Availability

The data that support the findings of this study are available from the corresponding author upon reasonable request. Data have been de-identified to protect participant confidentiality and are available subject to appropriate ethical approvals.

## Supplementary Materials

The following supporting information can be downloaded at: https://www.mdpi.com/article/10.3390/healthcare14010037/s1, Supplementary Material S1: Workshop 1 Zoom Whiteboard (Co-define); Supplementary Material S2: Workshop 2 face-to-face (Co-define); Supplementary Material S3: Workshop 3 Zoom Whiteboard (co-design); Supplementary Material S4: Think-aloud interview schedule (co-refine).

## Abbreviations

The following abbreviations are used in this manuscript:

COPD	Chronic Obstructive Pulmonary Disease
HCP	Healthcare Practitioner
UK	United Kingdom
