# Supplementary material for: Co-Creating a Digital Resource to Support Smartwatch Use in COPD Self-Management: An Inclusive and Pragmatic Participatory Approach
Source: Healthcare (Basel). 2025 Dec 23;14(1):37. doi: 10.3390/healthcare14010037 (PMC12785582; doi:10.3390/healthcare14010037)
Supplement: Supplementary file 1 [file healthcare-14-00037-s001.zip › healthcare-4042794-supplementary.pdf]

Supplementary Materials

Supplementary Material S1

Workshop 1 Zoom Whiteboard (Co-define)

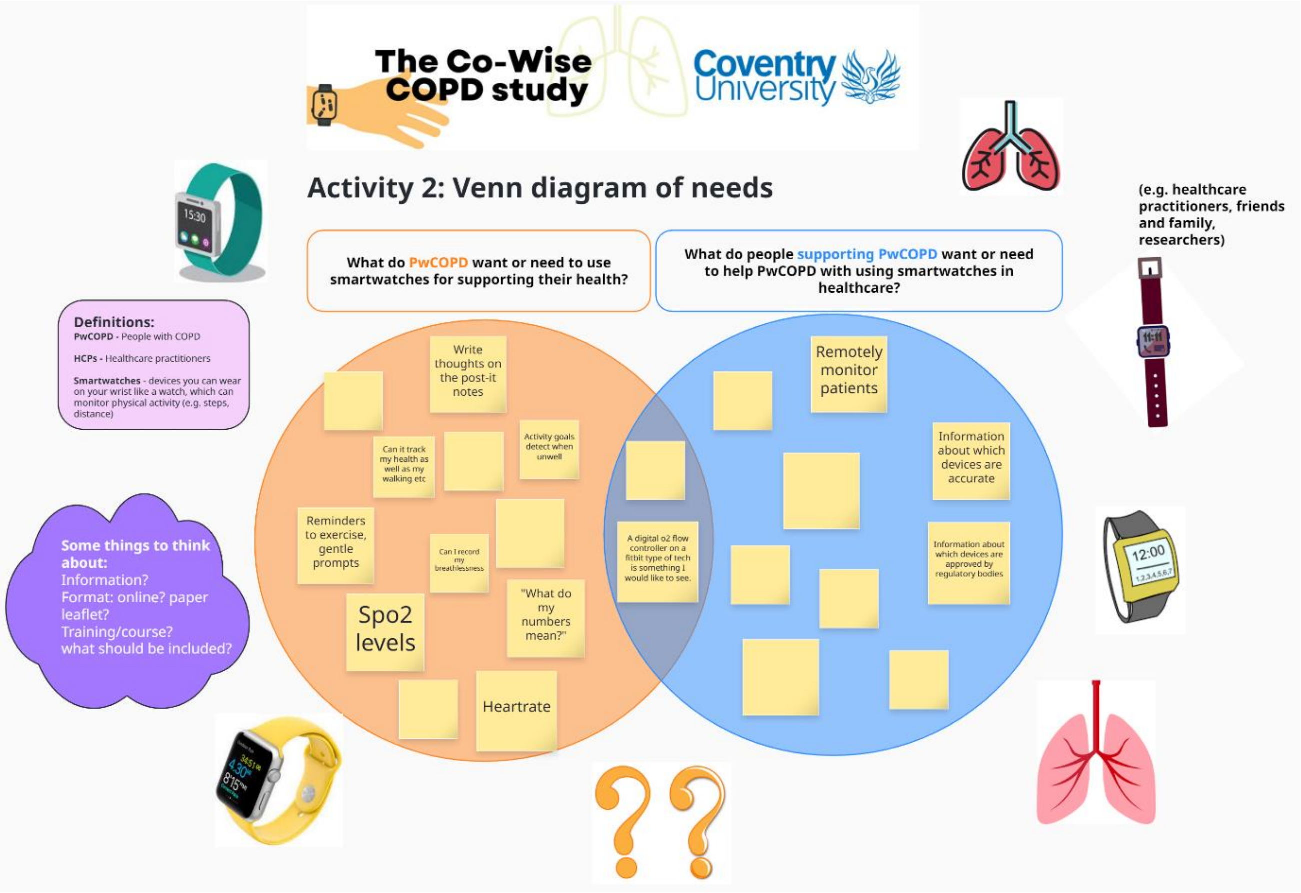

Supplementary Material S2

Workshop 2 face-to-face (Co-define)

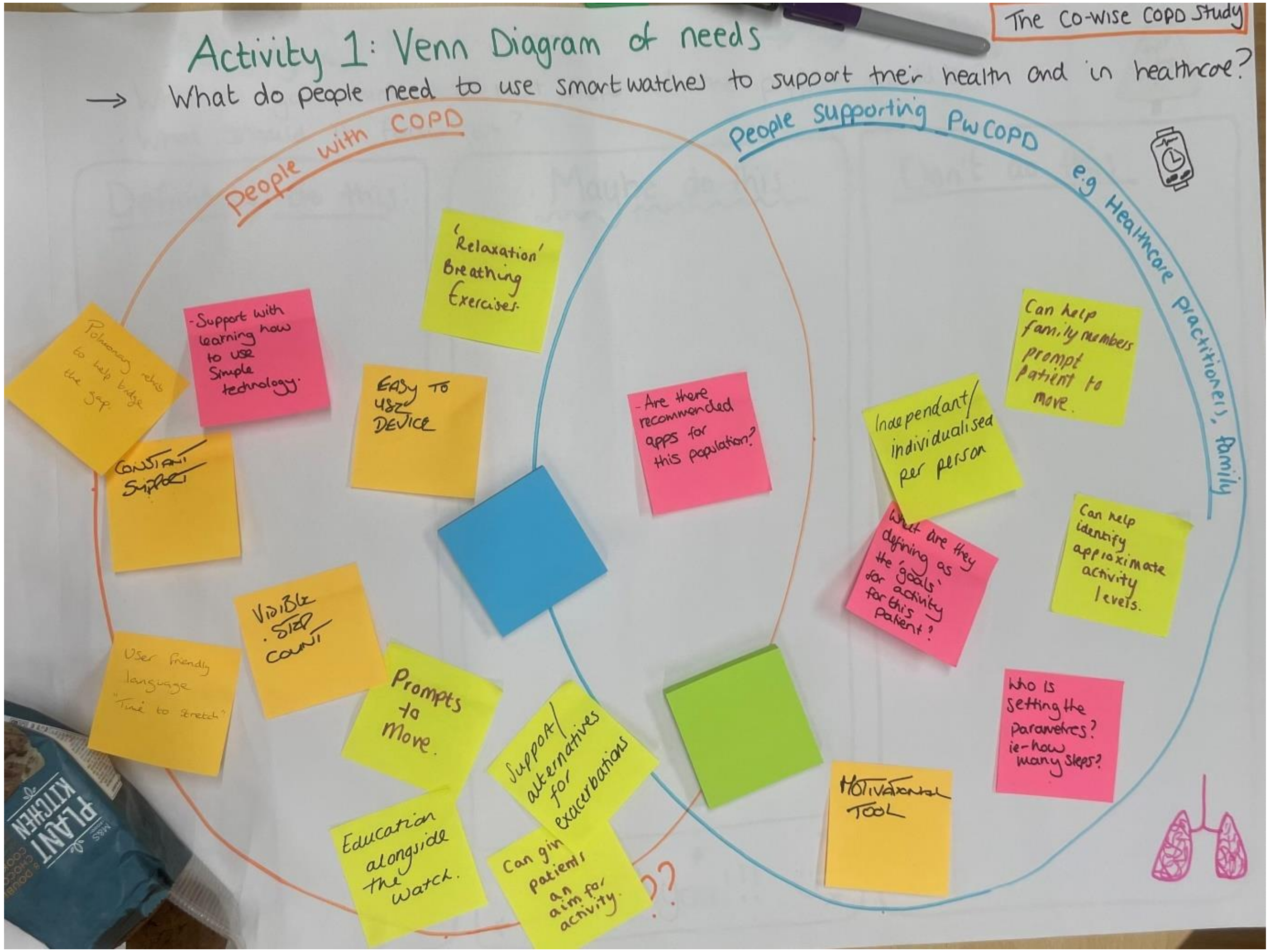

Supplementary Material S3

Workshop 3 Zoom Whiteboard (co-design).

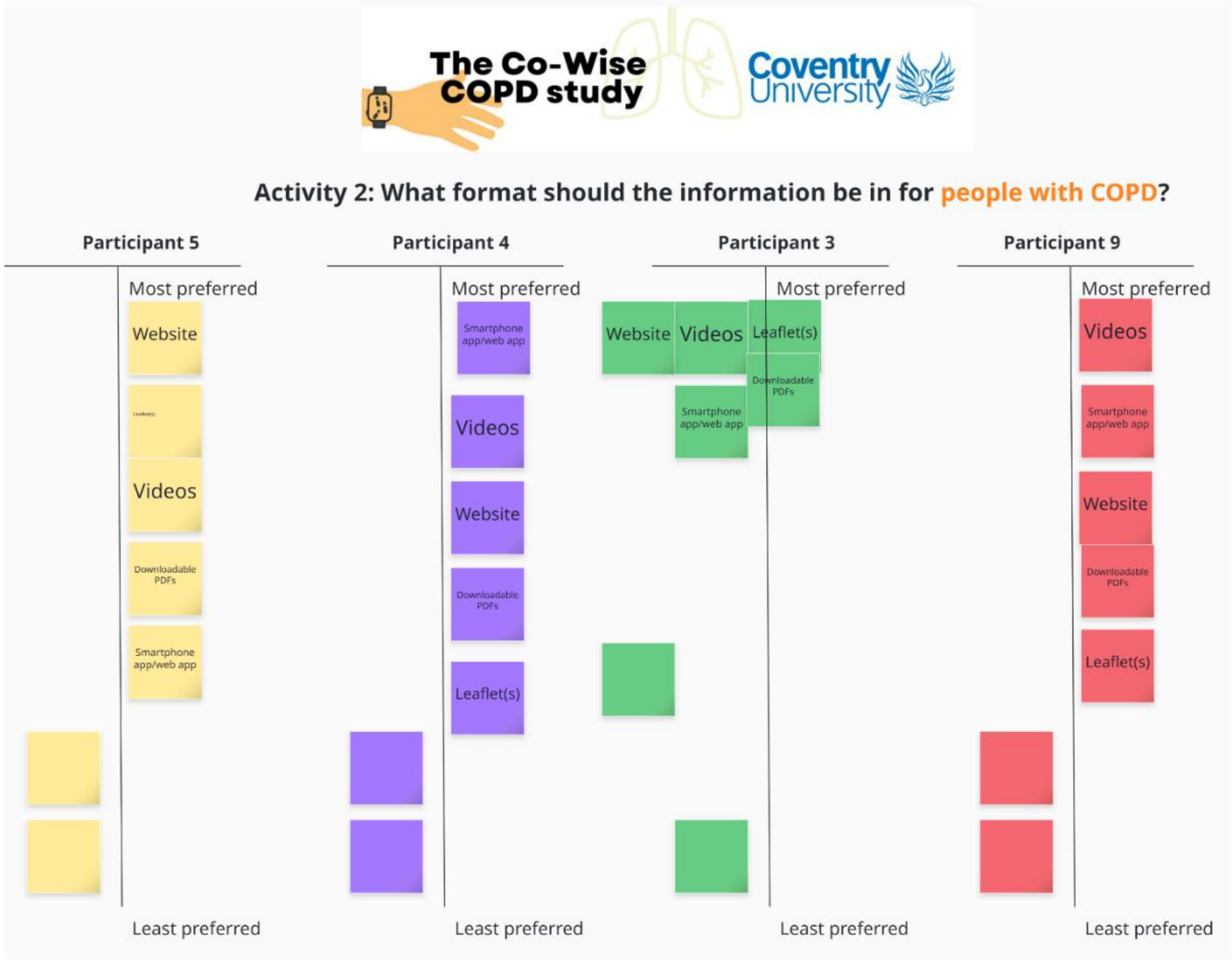

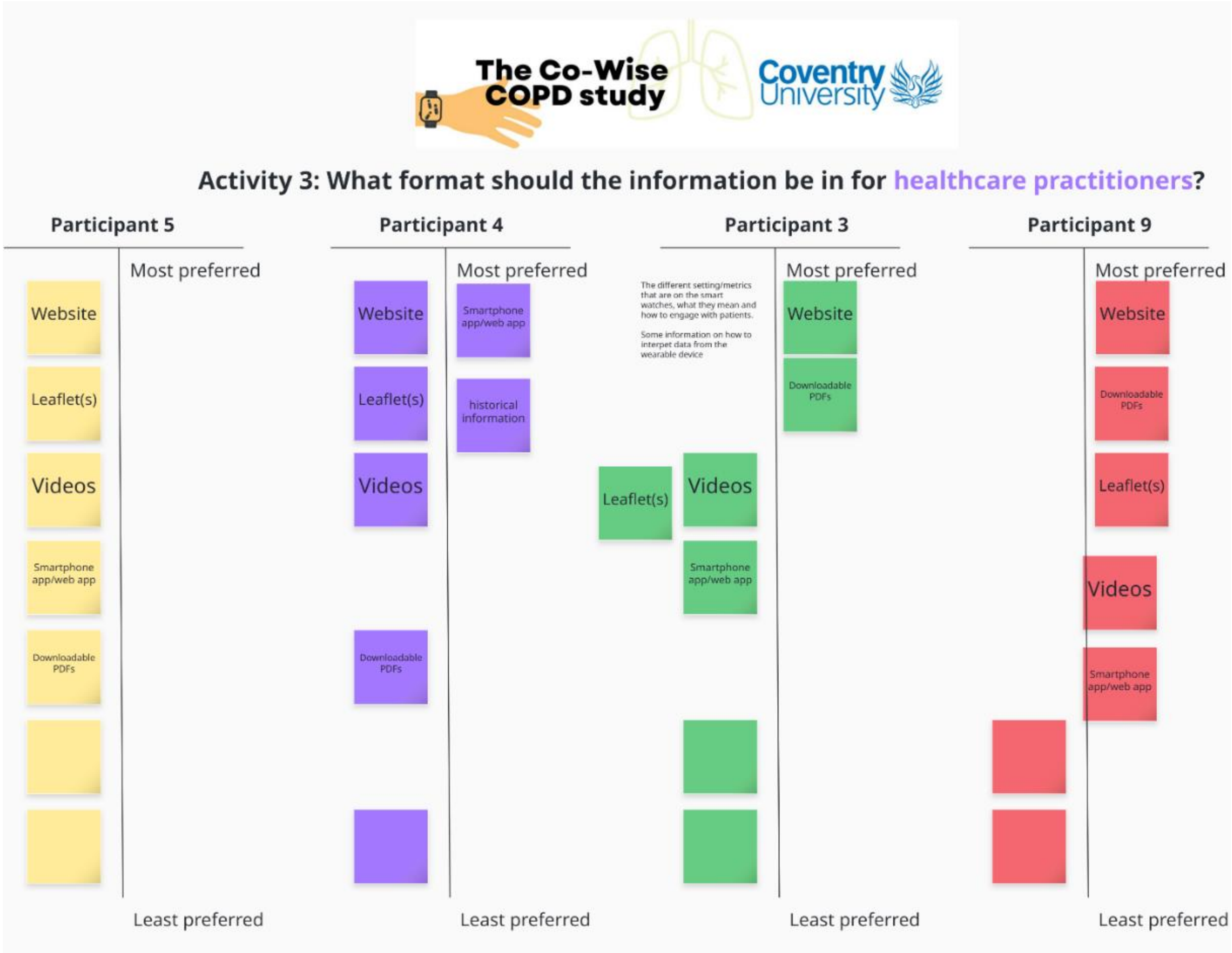
The Co-Wise COPD study
Coventry University
Activity 3: What format should the information be in for healthcare practitioners?
Participant 5
Most preferred
Website
Leaflet(s)
Videos
Smartphone app/web app
Downloadable PDFs
Least preferred
Participant 4
Most preferred
Website
Smartphone app/web app
Leaflet(s)
historical information
Videos
Downloadable PDFs
Least preferred
Participant 3
The different setting/metrics that are on the smart watches, what they mean and how to engage with patients.
Some information on how to interpet data from the wearable device
Most preferred
Website
Downloadable PDFs
Leaflet(s)
Videos
Smartphone app/web app
Least preferred
Participant 9
Most preferred
Website
Downloadable PDFs
Leaflet(s)
Videos
Smartphone app/web app
Least preferred

Supplementary Material S4

Think aloud interview schedule (Co-Refine)

- Thank you for arranging to meet for the Co-Wise COPD Study.
- The aim of this study is to co-create information for people with COPD and healthcare practitioners on using wearables for supporting self-management.
- I am interested in your thoughts about the website we have been creating, positive and negative, there are no right or wrong answers.
- Interviews will be recorded, transcribed, and analysed. All answers you give today will be anonymous and you will not be able to be identified within the results.
- This session will take approximately 1-hour minutes depending on how much you have to say.
- Any questions?
- Is it ok for me to record the interview, including the screen share?

Similar to last time (if previously done a think aloud), I would like you to tell me what you are thinking as you are going through.

The focus of today is for you to tell me what you think. If you've seen the information before, there might be some changes from the other interviews I have done, so feel free to read or look at any parts you are interested in.

There is no particular order I would like for you to look through, so use it as you would any other website (when they could go through the website).

As you are looking and reading through it would be really good for you to tell me what your thoughts are out loud, including anything that doesn't make sense, that you would change or think anything is missing.

Initial questions:

- What are your initial thoughts about this website?
- Is there anything you would like to add/change?

Specific aspects:

- What do you think about the pages?
- What do you think about the content?
- Is there anything that doesn't make sense?

- What are you thinking?
- Included an option to take part:
    - Would you click it?
    - What would you expect to see?

Format/ presentation?

- What do you think about:
    - The overall layout
    - Words
    - Language used
    - Content
    - Pictures
- Is there anything you would add/change?

HCP section – under development

- What would you expect to see in this section?
- What info do HCPs need?
- What are the barriers to those?
- What 'evidence' would you need to use this information?

Thank you

Any questions?
